# Sportomics method to assess acute phase proteins in Olympic level athletes using dried blood spots and multiplex assay

**DOI:** 10.1038/s41598-022-23300-y

**Published:** 2022-11-18

**Authors:** Adriana Bassini, Silvia Sartoretto, Lukas Jurisica, Alexandre Magno-França, Leigh Anderson, Terry Pearson, Morty Razavi, Vinod Chandran, LeRoy Martin, Igor Jurisica, L. C. Cameron

**Affiliations:** 1 Laboratory of Protein Biochemistry, Federal University of State of Rio de Janeiro, Av. Pasteur, 296 – Urca, Rio de Janeiro, R.J. 22290-350 Brazil; 2 SOmics, Vila Velha, ES Brazil; 3 School of Computer Science, Carleton University, Ottawa, Canada; 4 SISCAPA Assay Technologies, Inc., Washington, DC USA; 5 Arthritis Program, Schroeder Arthritis Institute, Krembil Research Institute, University Health Network, Toronto, ON Canada; 6 Faculty of Medicine, University of Toronto, Toronto, ON Canada; 7 Waters Technologies, Milford, MA USA; 8 Osteoarthritis Research Program, Division of Orthopedic Surgery, Schroeder Arthritis Institute, and Data Science Discovery Centre for Chronic Diseases, Krembil Research Institute, University Health Network, Toronto, Canada; 9 Departments of Medical Biophysics and Computer Science, and Faculty of Dentistry, University of Toronto, Toronto, ON Canada; 10 Institute of Neuroimmunology, Slovak Academy of Sciences, Bratislava, Slovakia; 11 Department of Computer Science, University of Toronto, Toronto, Canada

**Keywords:** Predictive markers, Immunochemistry, Mass spectrometry

## Abstract

Sportomics is a subject-centered holistic method similar to metabolomics focusing on sports as the metabolic challenge. Dried blood spot is emerging as a technique due to its simplicity and reproducibility. In addition, mass spectrometry and integrative computational biology enhance our ability to understand exercise-induced modifications. We studied inflammatory blood proteins (Alpha-1-acid glycoprotein—A1AG1; Albumin; Cystatin C; C-reactive protein—CRP; Hemoglobin—HBA; Haptoglobin—HPT; Insulin-like growth factor 1; Lipopolysaccharide binding protein—LBP; Mannose-binding lectin—MBL2; Myeloperoxidase—PERM and Serum amyloid A1—SAA1), in 687 samples from 97 World-class and Olympic athletes across 16 sports in nine states. Data were analyzed with Spearman's rank-order correlation. Major correlations with CRP, LBP; MBL2; A1AG1, and SAA1 were found. The pairs CRP-SAA1 and CRP-LBP appeared with a robust positive correlation. Other pairs, LBP-SAA1; A1AG1-CRP; A1AG1-SAA1; A1AG1-MBL, and A1AG1-LBP, showed a broader correlation across the sports. The protein–protein interaction map revealed 1500 interactions with 44 core proteins, 30 of them linked to immune system processing. We propose that the inflammation follow-up in exercise can provide knowledge for internal cargo management in training, competition, recovery, doping control, and a deeper understanding of health and disease.

## Introduction

Sportomics is a subject-centered holistic method, focusing on sports as the metabolic challenge (for an editorial and review, see Bragazzi, Bassini and Cameron)^1,2^. Unlike the studies carried out in the laboratory under extremely controlled conditions, our method involves samples that are collected in the field in an uncontrolled environment; thus, capturing important physiological changes during training and competition^3–5^.

Dried blood spot (DBS) is emerging as a collecting sample technique in several fields due to its simplicity and reproducibility^6,7^. Mass spectrometry in combination with integrative computational biology is enhancing our ability to study complex changes in health and disease with a patient-centered perspective^8,9^. Combined, these techniques can broaden the understanding of exercise-induced molecular changes at an individual athlete level.

After stressful exercise, Acute phase proteins (APPs) change in the blood^10^ and are related to overreaching and overtraining^6^. Understanding broad individual and combined response of APPs during acute phase response (APR) and signaling is an obvious way to study inflammation in exercise.

The uniqueness of metabolic stress caused by training and competition in top-level athletes can help us understand other types of hypermetabolic disorders including the ones that occur in diseases. Thus, it is critical that the scientific community access metabolic information on world-class competing top-level athletes^2^. Here we studied the inflammatory response in almost a hundred World-class and Olympic level athletes in 16 different sports and propose combined targets to understand exercise-induced modifications and training evaluation. We hypothesize that high intensity exercise can be an important model to study inflammatory response in hypermetabolic diseases.

## Methods

### Subjects

**Figure 1:**
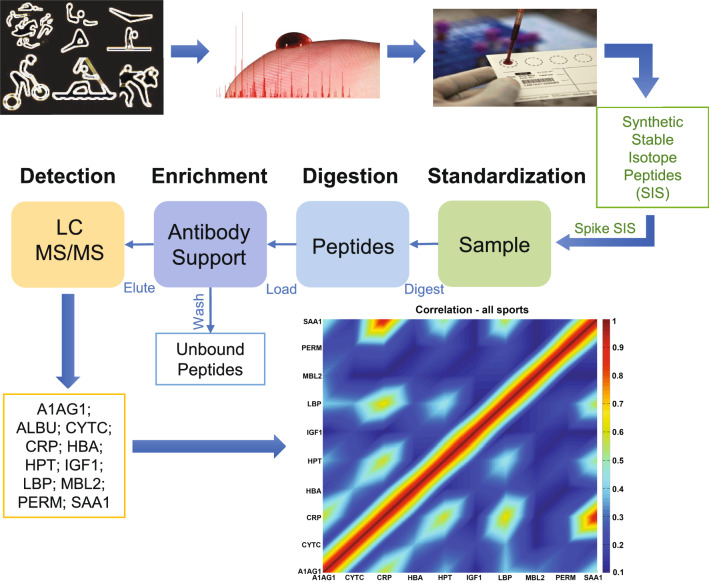
Experimental design. Analysis flowchart. Alpha-1-acid glycoprotein—A1AG1; Albumin—ALBU; Cystatin C—CYTC; C-reactive protein CRP; Hemoglobin beta chain—HBA; Haptoglobin—HPT; Insulin-like growth factor 1—IGF1; Lipopolysaccharide binding protein—LBP; Mannose-binding lectin—MBL2; Myeloperoxidase—PERM and Serum amyloid A1—SAA1.

Samples (687) were collected in the field in an uncontrolled environment at multiple time points during training and competition and in nine different states (fasting (eight hours minimum), re-hydration (rapid re-hydration after fasting and hydration deprivation), pre-, per-, or post-technical training; after physical training; pre- or post-competition and resting (60–90 min after either training or competition) from 97 athletes (men and women) across 16 different sports. Experimental study design is presented in Fig. 1. Our subjects were Olympic-level athletes who participated in various world championships, South American, Pan American, and Olympic Games. Most of them were podium medalists (Table 1). The research was approved by Ethical Committees (CAAE 0053.0.131.000-007; CAAE 2016.0.000.305-10 and 2.230.073). The study was performed in accordance with the ethical standards as laid down in the 1964 Declaration of Helsinki and its later amendments. An informed consent was obtained from the subjects, who were instructed as to the nature of the study and the procedures involved.

**Table 1:** Samples were collected in nine different states: fasting (eight hours minimum), re-hydration (rapid re-hydration after fasting and hydration deprivation), pre-, per-, or post-technical training; after physical training; pre- or post-competition and resting (60–90 min after either training or competition).

Sports	Samples		States								
					Training				World games		
	Total athletes	Total samples	Fasting	Re-hydration	Pre- training	Per- training	Post-training	After gym	Pre- competition	Post- competition	Resting
HOLO	97	687	48	5	184	2	197	8	106	89	48
Archery	6	6	6	0	0	0	0	0	0	0	0
Athletics	4	26	6	0	3	0	6	0	3	6	2
Basketball	12	48	0	0	24	0	24	0	0	0	0
Beach Volleyball	2	34	0	0	8	0	4	0	12	10	0
Boxing	7	16	1	4	0	0	0	0	11	0	0
Canoeing	4	58	8	0	12	2	16	8	0	0	12
Cycling	4	34	0	0	12	0	11	0	4	4	3
Diving	1	12	3	0	2	0	2	0	1	2	2
Gymnastics	8	84	0	0	25	0	22	0	17	18	2
Handball	15	139	1	0	27	0	31	0	41	37	2
Karate	7	63	1	1	28	0	23	0	5	3	2
Modern Pentathlon	5	111	21	0	24	0	39	0	0	4	23
Softball	14	38	0	0	14	0	14	0	10	0	0
Swimming	3	3	0	0	0	0	0	0	0	3	0
Taekwondo	3	7	1	0	1	0	3	0	2	0	0
Racewalking	2	8	0	0	4	0	2	0	0	2	0

HOLO refers to all combined, holistic samples and analysis.

### Samples

Capillary blood dry blood spot (DBS) samples were collected at multiple time points using a Sportomics approach^1^. Samples were collected using lancet finger-pricks (Medlance Plus Extra or Special, HTL Strefa; Medline, Cat. No. HTD7045BX) dried on Whatman 903 Protein Saver DBS cards. The cards were dried at 4 °C in the presence of a desiccant and processed on a daily basis. The samples were collected during the PanAm games in Toronto, ON, Canada (July 7th-26th 2015) and were shipped on a daily basis to the lab located in Victoria, BC, Canada. More specifically, the samples were processed between July 11th and July 22nd 2015.

### SISCAPA-LC-MRM protein measurement

A panel of 11 proteins of known clinical significance (Alpha-1-acid glycoprotein—A1AG1; Albumin—ALBU; Cystatin C—CYTC; C-reactive protein CRP; Hemoglobin beta chain—HBA; Haptoglobin—HPT; Insulin-like growth factor 1—IGF1; Lipopolysaccharide binding protein—LBP; Mannose-binding lectin—MBL2; Myeloperoxidase—PERM and Serum amyloid A1—SAA1) was measured using SISCAPA-MRM mass spectrometry as described^11^.

For each peptide in each sample, the primary data comprises the ratio (peak area ratio, or PAR) of the area of a sample-derived proteotypic tryptic peptide to the area of an added synthetic stable-isotope-labeled version of the same peptide sequence serving as an internal standard. Identical amounts of each internal standard were added to each trypsin-digested sample, allowing direct comparison of the amounts of each protein between samples (Table 2).

**Table 2:** Eleven blood proteins were measured using SISCAPA immuno-affinity enrichment mass spectrometry.

Protein	Entry name	Short protein name	Gene name	ID	Isoform	Peptide
Alpha-1-acid glycoprotein	A1AG1	AGP 1	OMR1	P02763	1	NWGLSVYADKPETTK
Albumin	ALBU	Albumin	ALB	P02768	1 & 3	LVNEVTEFAK
Cystatin C	CYTC	Cystatin-C	CST3	P01034	1	ALDFAVGEYNK
C-reactive protein	CRP	C-reactive protein	CRP	P02741	1 & 2	ESDTSYVSLK
Hemoglobin beta chain	HBA	Hemoglobin subunit alpha	HBA1, HBA2	P69905	1	VHLTPEEK
Haptoglobin	HPT	Haptoglobin	HP	P00738	1 & 2	VTSIQDWVQK
Insulin-like growth factor 1	IGF1	IGF-I	IGF1	P05019	all	GFYFNKPTGYGSSSR
LPS-binding protein	LBP	LBP	LBP	P18428	1	LAEGFPLPLLK
Mannose-binding lectin	MBL2	MBP-C	MBL2	P11226	1	EEAFLGITDEK
Myeloperoxidase	PERM	MPO	MPO	P05164	all	DYLPLVLGPTAMR
Serum amyloid A1	SAA1	SAA	SAA1	P0DJI8	1	GPGGVWAAEAISDAR

### Data analysis

PAR data were assembled in Tableau Prep Builder (Tableau Software, Inc., Seattle WA, USA) and joined with tables defining sample characteristics (e.g., date of collection, subject contextual health notes, SIS concentrations, analytical run structures, etc.). Data analysis and visualization were performed in Tableau Desktop (Tableau Software, Inc., Seattle, WA, USA).

To minimize the effect of variations in plasma volume between DBS from the same individual (typically ± 10–15%), we refined and applied a simplified version of a method developed previously to normalize plasma volume by computing the ratio of each protein to that of albumin (a protein whose abundance is usually very stable within individuals over time (17) and then dividing by the individual’s average value of the protein (to put measurements on a personalized scale of relative change between samples).

Replicate DBS samples were processed for SISCAPA measurement. The average coefficient of variation (CV) across the assays used in Datasets was 10%, consistent with expectations for clinically useful assays.

### Bioinformatics analysis

Raw data from SISCAPA platform were analyzed using SciPy Python library (ver. 1.4.1). We used non-parametric Spearman's rank-order correlation (r_s_, scipy.stats.spearmanr method). The diagonal entries are self-correlations, and the off-diagonal entries represent direct positive or negative correlations of pairwise proteins (the correlation matrix is symmetric). Supplementary Material 1 presents all data, but further discussion focuses only on protein pairs with r_s_ > 0.5 and significance of *p* < 10^–3^.

Data were visualized using Matlab R2019b (Mathworks, Natick, MA) and the PyPlot Python library (ver. 3.1.2). To emphasize correlations with statistical significance, only correlations with *P* value < 0.01 were used in the resulting image. The color of each cell was then determined by linearly interpolating the correlation (blue for positive correlation, red for negative correlation).

PPI map was created by querying IID database version 2021^12^ with 11 proteins, and obtaining all their physical interactions, using default setting and all sources (http://ophid.utoronto.ca/iid). Resulting interaction network was visualized using NAViGaTOR ver. 3.0.16^13^, and the final figure with legends was prepared in Adobe Illustrator ver. 26.6 from the SVG file.

## Results

**Figure 2:**
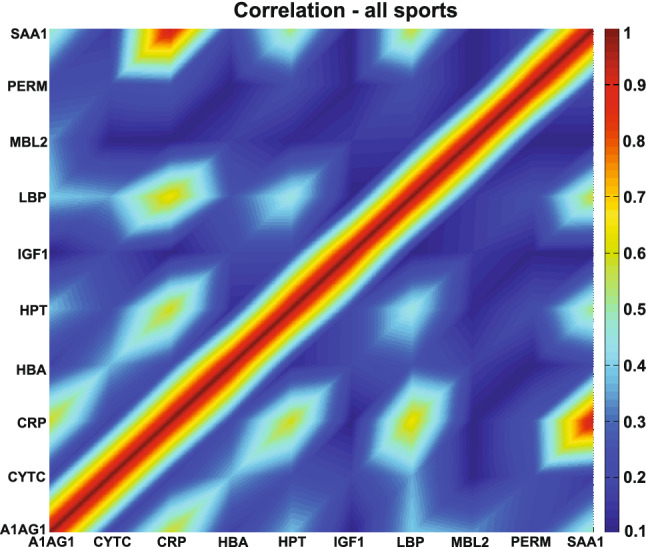
CRP and LBP and CRP and SAA1 are strongly correlated protein pairs. Matrix of pairwise correlations among proteins, considering all sports, athletes, and states (HOLO). The absolute value of correlations is considered. Spearman correlation was used, and no p-value filter was applied. Full data are presented in Supplementary Material 1. Alpha-1-acid glycoprotein—A1AG1; Albumin—ALBU; Cystatin C—CYTC; C-reactive protein CRP; Hemoglobin beta chain—HBA; Haptoglobin—HPT; Insulin-like growth factor 1—IGF1; Lipopolysaccharide binding protein—LBP; Mannose-binding lectin—MBL2; Myeloperoxidase—PERM and Serum amyloid A1—SAA1.

We measured and analyzed 11 blood proteins in 687 samples from 97 athletes across 16 sports in nine different states (Table 1 and Fig. 1). To obtain a holistic view of the proteins' presence in the blood across the states in various sports, we correlated all the analyzed proteins during all states, athletes, and sports (Holistic, HOLO). It is known that CRP and SAA1 positively correlate during acute inflammation^8^, and our HOLO analysis confirms it across all sixteen sports, 97 athletes and nine states, in addition to identifying CRP-LBP positive correlation (Fig. 2).

**Figure 3:**
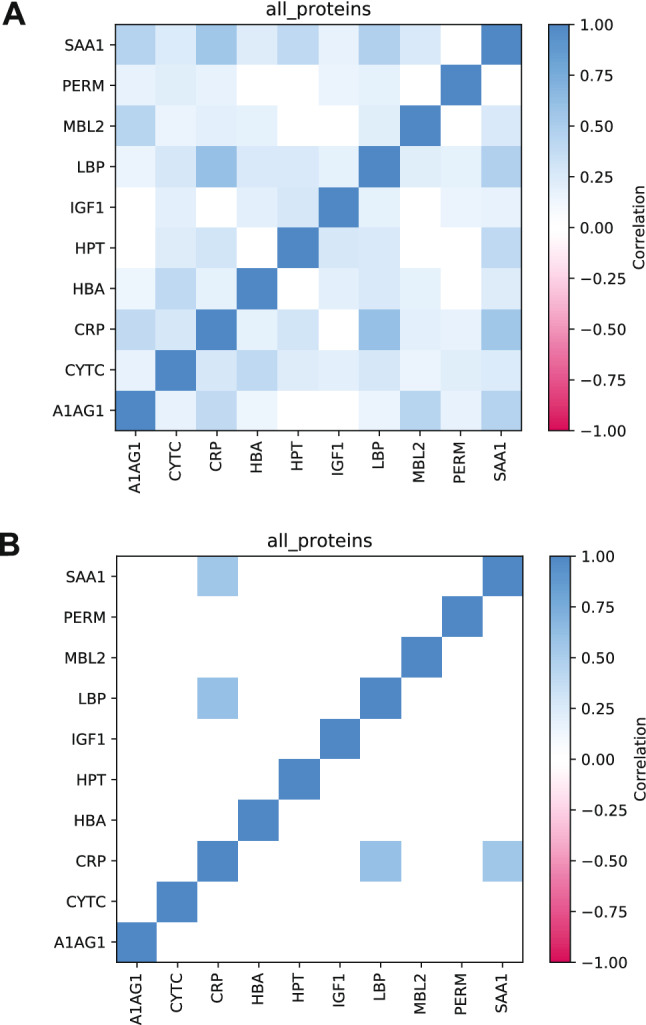
There are two high protein pair correlations *p* < 0.001 and r_s_ > 0.5. Matrix of pairwise correlations among proteins, considering all sports, athletes and states. (**A**) Significant protein pairs correlations at *p* value < 0.001 are highlighted; (**B**) Significant protein pairs correlations r_s_ > 0.5 are highlighted (*p* < 0.001). n = 97. Full data are presented in Supplementary Material 1. Alpha-1-acid glycoprotein—A1AG1; Albumin—ALBU; Cystatin C—CYTC; C-reactive protein CRP; Hemoglobin beta chain—HBA; Haptoglobin—HPT; Insulin-like growth factor 1—IGF1; Lipopolysaccharide binding protein—LBP; Mannose-binding lectin—MBL2; Myeloperoxidase—PERM and Serum amyloid A1—SAA1.

We choose to discuss significant protein correlations with a p < 0.001 (comprehensive results at other p-value cutoffs—*p* < 0.001 to *p* < 0.05—are shown in the Supplementary Material 1) due to the diversity of the analyzed data. We found two significant protein correlations when analyzing HOLO: CRP-LBP r_s_ = 0.60 (*p* < 10–9) and CRP-SAA1 r_s_ = 0.56 (*p* < 10–9) (Fig. 3).

**Figure 4:**
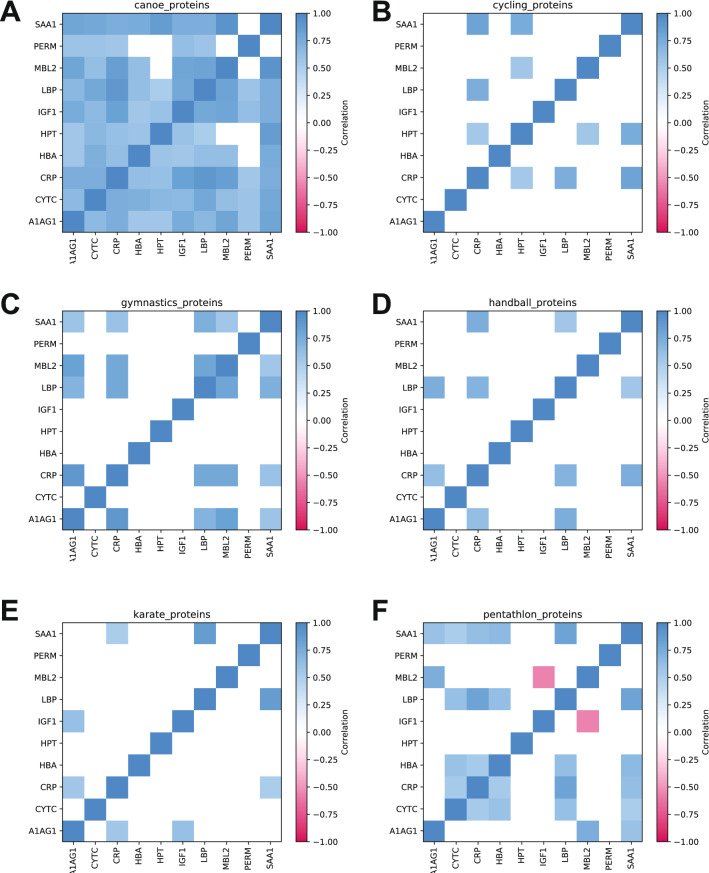
SAA1 and CRP are strongly correlated. LBP and CRP are also strongly correlated. Matrix of pairwise correlations among proteins for (**A**) canoeing (n = 58); (**B**) cycling (n = 34); (**C**) gymnastics (n = 84); (**D**) handball (n = 139); (**E**) karate (n = 63) and (**F**) modern pentathlon (n = 111). The positive (blue) and negative (red) correlations at *p* value < 0.001 are shown. Protein correlations r_s_ > 0.5. Full data are presented in Supplementary Material 1. Alpha-1-acid glycoprotein—A1AG1; Albumin—ALBU; Cystatin C—CYTC; C-reactive protein CRP; Hemoglobin beta chain—HBA; Haptoglobin—HPT; Insulin-like growth factor 1—IGF1; Lipopolysaccharide binding protein—LBP; Mannose-binding lectin—MBL2; Myeloperoxidase—PERM and Serum amyloid A1—SAA1.

To characterize protein correlation across individual sports and states, we selected sports with sufficient number of athletes (n ≥ 4) and time points (i.e., different collection at different states; m ≥ 8 on average). Six major sports satisfied the criteria, and as could be expected, the correlation pattern differed across sports. CRP and SAA1 were strongly correlated in all six sports, as seen for HOLO correlation matrices. Also, CRP and LBP showed strong correlation in all individual sports except for karate (Fig. 4A–F).

All the canoeing athletes had a same strict nutrition regimen, training, and rest. This particular system allows us to have a close and strict collection protocol, decreasing variability. We measured proteins from four male athletes for two years and six states. Canoeists’ protein analysis revealed 40 significant protein pairs, all of these with r_s_ > 0.50 (Fig. 4A).

The analyzes of cyclists' proteins shows a high positive correlation between five pairs of proteins (Fig. 4B). Ten protein pairs were correlated in gymnasts (Fig. 4C). Handball was the only team sport analyzed with five pairs of proteins correlated, while in the combat sport karate, four pairs of proteins were associated in our study (Fig. 4D and E).

Many collections in different and variable states were performed in modern pentathlon due to our Sportomics approach. We collected samples and measured proteins from five athletes (four female) for two years and five different states. The analyzes from the evaluated proteins correlations in modern pentathlon athletes exhibited 12 pairs. Interestingly, the IGF1-MBL2 pair exhibited a negative correlation (Fig. 4F).

**Figure 5:**
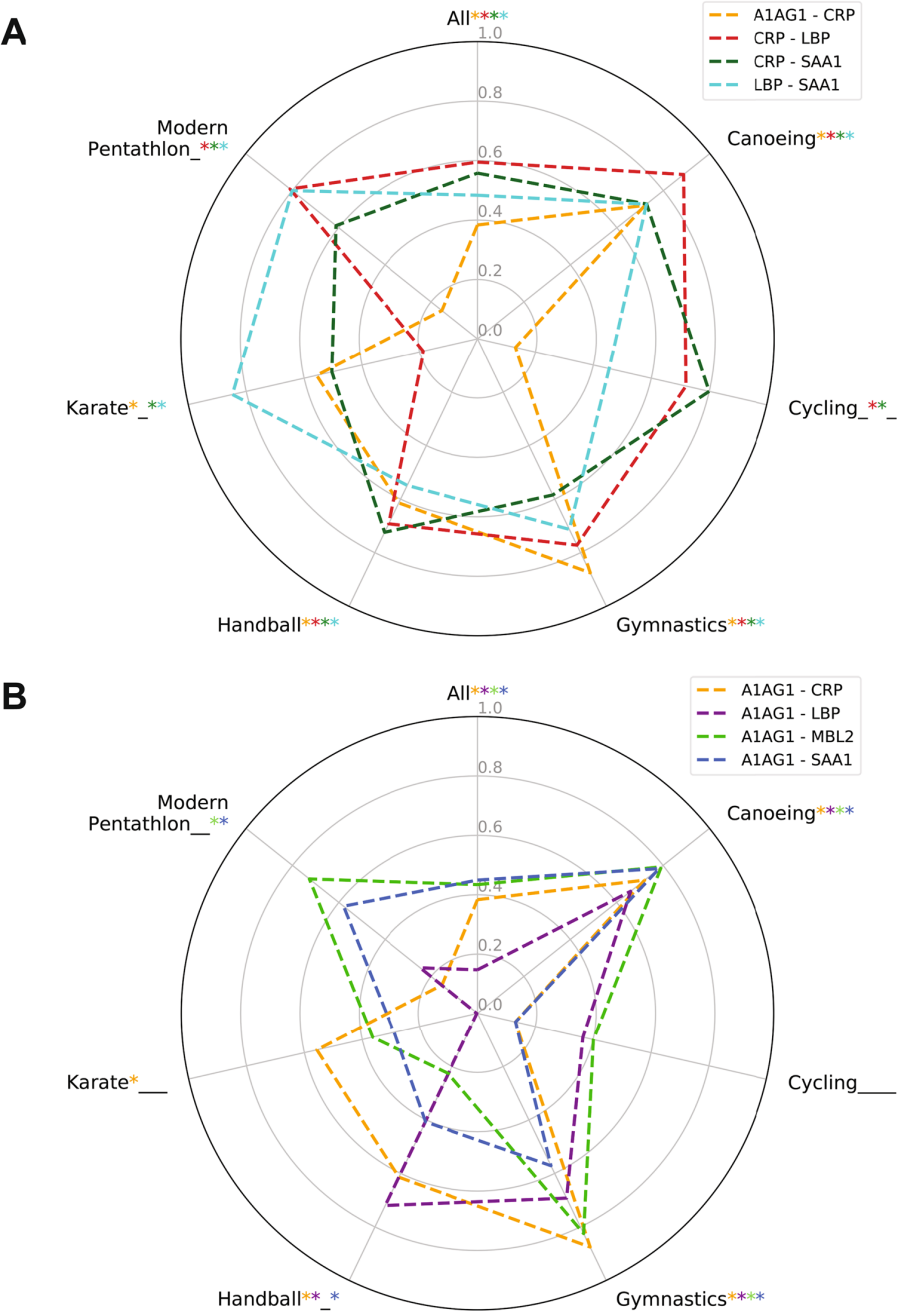
Seven proteins correlated in three or more sports. Matrix of pairwise correlations (r_s_) among proteins for canoeing; cycling; gymnastics; handball; karate, and modern pentathlon. The significant correlations at *p* value < 0.001 are shown (*). Full data are presented in Supplementary Material 1. Alpha-1-acid glycoprotein—A1AG1; Albumin—ALBU; Cystatin C—CYTC; C-reactive protein CRP; Hemoglobin beta chain—HBA; Haptoglobin—HPT; Insulin-like growth factor 1—IGF1; Lipopolysaccharide binding protein—LBP; Mannose-binding lectin—MBL2; Myeloperoxidase—PERM and Serum amyloid A1—SAA1.

To interpret changes of correlated protein pairs across sports better, we focused on the most varying seven pairs (Fig. 5). The positive control—CRP-SAA1—is confirmed in all six studied sports and HOLO (r_s_: 0.56–0.80) (Fig. 5A). CRP-LBP correlated in five of the six selected sports and HOLO (r_s_: 0.59 to 0.89; Fig. 5B). Other protein pairs show a wider correlation across the sports: LBP-SAA1 (r_s_: 0.45–0.85); A1AG1-CRP (r_s_: 0.13–0.87); A1AG1-SAA1 (r_s_: 0.13–0.78); A1AG1-MBL2 (r_s_: 0.40–0.82) and A1AG1-LBP (r_s_: 0.1–0.72) (Fig. 5A and B).

Four proteins (HBA; HPT; MBL2 and SAA1) did not correlate (i.e., r_s_ > 0.5) with PERM in any of the six major sports of our study (Supplementary Material 1).

**Figure 6:**
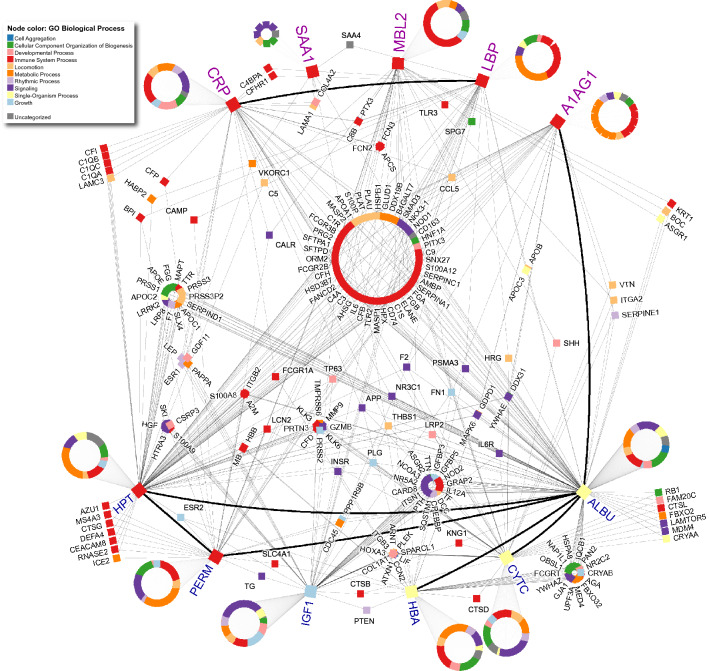
Thirty high connectivity core immune-processing proteins were found according to our PPI Map. The described human protein interactions were analyzed and mapped. A detailed list of 1500 protein interactions can be found in the Supplementary Material 2.

The protein–protein interaction map (PPI) revealed 1500 protein interactions with 44 high connectivity core proteins, 30 of them linked to immune system processing (Fig. 6).

## Discussion

Exercise can be used as a model for the understanding of different physiological and physiopathological responses. Our study used an innovative multiplex method for the simultaneous measurement of selected inflammatory proteins associated with the sportomics approach. Sportomics is an ex post facto design and does not attempt to control the variables but rather to understand them^1,2,14–20^. The uniqueness of this study is the fact that all 97 athletes analyzed belong to an elite group of world top-caliber competitors in their respective sports disciplines. Most of them are medalists not only in World Championships and Olympic Games but also in several editions of other multi-sport events such as the World Military Games, the Pan American Games, and the South American Games.

Due to the large number of variables in the study, we focused on correlations r_s_ > 0.5 (Spearman's rank correlation) with a significance of *p* < 0.001. We acknowledge that significant results with low correlations can still be important. We use stringent cutoffs in our study due to its observational nature, since we report findings from in the field measurements across diverse events as metabolically different as intense training with recovery. However, even small changes in a protein expression or its correlation with another protein can represent biologically meaningful signal.

The highest number of correlated protein pairs in our study appeared in canoeing. We studied the athletes' characteristics in a previous study^19^. Due to being confined in a small training center, the four canoeing athletes in our study had a strong similarity in hydration, nutrition, training, competition, and rest. And more, they are all sprint canoeists competing exactly in the same distances, either sharing the same boat in pairs either paddling alone. A less controlled group, the MP athletes, exhibited 12 protein correlation pairs.

We have previously shown that CRP and SAA1 strongly correlate during a week of training of Olympic level beach volleyball athletes^8^. In this study, CRP-SAA1 appeared with a significant and robust positive correlation even when analyzed in HOLO. CRP-SAA1 remained highly correlated even at the individual sport level analysis. Several studies have investigated the functions of SAA1, including chemotaxis, the induction of macrophages, chemokines, and cytokines^21,22^. Additionally, CRP is related to many immune pathways, such as the signaling of inflammation^23^. It is important to emphasize that our subjects belong to 16 different sports, varying from individual to collective sports, cyclic or acyclic exercises of different intensities and volumes. CRP and SAA1 are positive APPs, and their correlation has been described in different physical conditions, including infection, trauma, inflammatory reactions, and hypermetabolic diseases, including prognosis of COVID-19^24–27^. The protein pair seems to be correlated to either increased inflammatory stress caused by acute exercise^28,29^ or its decrease caused by regular training in some diseases^30^.

Lipopolysaccharide-binding protein (LBP) is emerging in importance in understanding and diagnosing the inflammatory acute phase response (APR). It has been shown that the blood concentration of LBP can rise up to 40-fold in response to inflammatory stress in an interleukin-1 (IL-1) and IL-6 mediated response^31^. LBP synthesis and binding to lipopolysaccharide (LPS) can trigger a Janus response due to either the protein's pro-inflammatory effect in LPS presentation to CD-14 or the anti-inflammatory effect due to the LBP ability in transferring LPS to lipoproteins plasmatic and other mechanisms^32^. In addition to either microbiota or intestinal integrity, other different environmental stimuli seem to be associated with LBP response^33^. A very-low-calorie ketogenic diet, training history and heat are associated with changes in LBP^34–36^. While poorly studied in exercise, a previous study showed that poor physical function correlates with the increase in both CRP and LBP^37^. Our data revealed that LBP has a highly correlated positive response with CRP, analogously to SAA1. These results corroborate our previous study, where both CRP-SAA1 and CRP-LBP are highly correlated during a holistic analysis of inflammation that included physiological or physiopathological and exercise inflammatory responses^8^. The reduction of TLR4, the LPS/LBP complex receptor, seems to be related to exercise's protective effect in heart diseases^38^. Elucidating the response of LBP in exercise can help us understand the paradoxical effects of exercise that either triggering or decreasing the inflammatory response.

The pair LBP-SAA1 was highly correlated across five of the six individual sports except cycling, and when all 16 sports were combined (r_s_ < 0.5). It has been shown that LBP concentrations are directly related to LPS^39^. LPS is recognized by SAA1 which promotes LPS clearance and suppressing LPS‐induced inflammation^40^. LPS directly stimulates T helper 17 lymphocytes (Th17) by way of its highly expressed toll-like receptor-4 (TLR4)^41^. LPS entry in the blood is a recognized way of evaluating intestinal permeability (IP)^42^. The increase of both volume and intensity training is linked to IP and escalation in bacterial permeability to blood^43^. Furthermore, IP emerged as a target for elite athletes to follow up^44^. SAA1 is a mediator of local effector Th17 response driven by the gut microbiome. An unregulated response of Th17 is related to auto-inflammatory diseases^45–47^. So, understanding the LBP-SAA1 pairing in our system can help us to elucidate the role of inflammation in both exercise and disease.

Since the originals studies of Albert Szent-Györgyi of actin and myosin interaction, PPIs have exponentially grew in both coverage and annotation, and enabled systems biology^48^. Our PPI map reveals many possible targets for future investigation in both exercise science and inflammatory response. The central core in our PPI network reveals 30 proteins that can be of interest. For example, SERPINE family proteins as SERPINC1 and SERPINA1 appeared in our map and should be further investigated. In our study, the A1AG1 paired with four other proteins: CRP, LBP, MBL2, and SAA1. Our knowledge regarding the relationship of the mechanisms of the response of A1AG1 in exercise remains limited. A1AG1 can act as a binding and carrying protein for several known molecules. As an APP can increase up to six-fold during an inflammatory event, so understanding the function of the protein as an APP is essential for the knowledge of both pharmacodynamics and pharmacokinetics^49^. In addition to glucocorticoids, TNFα, IL-1, and IL-6, A1AG1 is regulated by other APPs as CRP, haptoglobin (HPT), SAA1, and hemopexin^50^. Important roles for A1AG1 include its ability to regulate angiogenesis and acting as anorexigenic via the leptin pathway^51,52^. It was demonstrated that A1AG1 rises glucose uptake in adipocytes, increasing both muscle glycogen content and muscle performance via the C–C chemokine receptor type 5, delaying the manifestation of fatigue^53–55^. It is proposed that A1AG1 can act as an immune-modulator of APPs via direct interaction with LPS^56^. The association of A1AG1 with CRP, SAA1, and LBP was already shown in a previous proteomic study where the authors used CRP as a control to establish the effectiveness of their method^57^. Similarly, we propose that other correlations found by our analyses deserve further investigations in controlled conditions and homogeneous cohorts.

PERM does not correlate with four proteins HBA, HPT, MBL2, and SAA1 in any of the six sports. Myeloperoxidase (PERM) is a peroxidase mainly expressed in neutrophils that rises in response to exercise, overreaching and overtraining^6,8,58^. We have previously shown that neutrophils count increase together with CRP in high-intensity exercise^3^. In another high-intensity exercise study, we showed that neutrophils' increase is not necessarily related to changes in red blood cells (RBC) increase^4^. Thus, the lack of correlation regarding HBA-PERM may be due to the increased blood neutrophils not correlated with RBC, and subsequently, HBA changes. Previous studies have shown that SAA1 produced by the intestine acts as a systemic signal to restrict aberrant activation of neutrophils, enhancing their ability to migrate to wounds^59–61^. It is possible to propose that the lack of PERM and SAA1 correlation could be due to a protective effect of SAA1 diminishing the neutrophil intestinal local response and regulating its migration to exercise-wounded tissue.

The use of exercise as a model of systems biology studies is well established^3–5^. The idea that exercise-induced inflammation is equivalent to the one that occurs in general medical and surgical conditions is not new^62^. It has been proposed that "metabolic sequelae of sustained exercise are similar, but not analogous, to the acute-phase response"^63^. Exercise can either improve or impair immunity depending on the volume and intensity and can be an excellent approach for studying inflammatory response^64^. The inflammation follow-up in exercise will provide knowledge for internal cargo management in training, competition, recovery, and doping control^14,17,65,66^, as well as a deeper understanding of inflammation in health and disease.

## Conclusions

Sportomics combined with dried blood spot and multiplex mass spectrometry can be used to understand exercise-induced systemic changes at an individual athlete level. Our study analyzes 11 blood proteins in 687 samples from 97 elite athletes across 16 sports in nine different states and showed that five acute phase proteins were highly correlated. Our protein–protein interaction map highlights 1500 protein–protein interactions (PPIs) with 44 core proteins, 30 of which are linked to immune system processing.

Future trials should explore the correlation pairs and PPIs emerging in our study as suitable markers of inflammation for understanding disease and exercise management. The follow-up of inflammation response triggered by exercise may provide knowledge for exercise internal cargo management in training, competition, recovery, doping control, and a deeper understanding of inflammation processes in health and disease.

## Supplementary Information

Supplementary Information 1.

Supplementary Information 2.

## Data Availability

All data generated or analyzed during this study are included in this published article [and its Online Resources files].
